# Supplementary material for: Multi‐dataset identification of innovative feature genes and molecular mechanisms in keratoconus
Source: J Cell Mol Med. 2024 Sep 19;28(18):e70079. doi: 10.1111/jcmm.70079 (PMC11412914; doi:10.1111/jcmm.70079)
Supplement: Supplementary file 2 — Material S2. [file JCMM-28-e70079-s002.zip › 7.GSEA analysis/6.SNORA74B/KEGG_ADIPOCYTOKINE_SIGNALING_PATHWAY.html]

Details for gene set KEGG\_ADIPOCYTOKINE\_SIGNALING\_PATHWAY[GSEA]

|  || Dataset | SNORA74B.group.cls#h\_versus\_l |
| Phenotype | group.cls#h\_versus\_l |
| Upregulated in class | l |
| GeneSet | KEGG\_ADIPOCYTOKINE\_SIGNALING\_PATHWAY |
| Enrichment Score (ES) | -0.38688186 |
| Normalized Enrichment Score (NES) | -1.3601321 |
| Nominal p-value | 0.13545817 |
| FDR q-value | 0.3316097 |
| FWER p-Value | 0.925 |
Table: GSEA Results Summary

  

Fig 1: Enrichment plot: KEGG\_ADIPOCYTOKINE\_SIGNALING\_PATHWAY      
 Profile of the Running ES Score & Positions of GeneSet Members on the Rank Ordered List

  

| SYMBOL | TITLE | RANK IN GENE LIST | RANK METRIC SCORE | RUNNING ES | CORE ENRICHMENT || 1 | LEP | na | 1168 | 0.461 | 0.0095 | No |
| 2 | ADIPOQ | na | 2104 | 0.362 | 0.0165 | No |
| 3 | CAMKK1 | na | 2124 | 0.360 | 0.0496 | No |
| 4 | PRKCQ | na | 5170 | 0.220 | -0.0176 | No |
| 5 | IRS4 | na | 6111 | 0.189 | -0.0270 | No |
| 6 | IKBKB | na | 8763 | 0.119 | -0.0924 | No |
| 7 | MTOR | na | 8938 | 0.115 | -0.0866 | No |
| 8 | AKT2 | na | 9380 | 0.105 | -0.0895 | No |
| 9 | CPT1A | na | 9758 | 0.097 | -0.0912 | No |
| 10 | AGRP | na | 9987 | 0.093 | -0.0891 | No |
| 11 | MAPK9 | na | 10427 | 0.085 | -0.0939 | No |
| 12 | PRKAG2 | na | 10916 | 0.076 | -0.1008 | No |
| 13 | STK11 | na | 10951 | 0.076 | -0.0947 | No |
| 14 | ACSL1 | na | 11987 | 0.060 | -0.1189 | No |
| 15 | CPT1B | na | 12076 | 0.059 | -0.1159 | No |
| 16 | RXRA | na | 12303 | 0.056 | -0.1172 | No |
| 17 | ADIPOR1 | na | 13284 | 0.044 | -0.1414 | No |
| 18 | PTPN11 | na | 13916 | 0.037 | -0.1561 | No |
| 19 | PPARA | na | 14290 | 0.033 | -0.1638 | No |
| 20 | TRADD | na | 14758 | 0.027 | -0.1748 | No |
| 21 | MAPK10 | na | 14996 | 0.024 | -0.1793 | No |
| 22 | PRKAG3 | na | 15089 | 0.024 | -0.1797 | No |
| 23 | TRAF2 | na | 15165 | 0.023 | -0.1798 | No |
| 24 | ACACB | na | 15478 | 0.020 | -0.1869 | No |
| 25 | CHUK | na | 16402 | 0.010 | -0.2126 | No |
| 26 | PRKAG1 | na | 17244 | 0.002 | -0.2366 | No |
| 27 | AKT1 | na | 18366 | -0.010 | -0.2680 | No |
| 28 | PRKAA1 | na | 18783 | -0.015 | -0.2786 | No |
| 29 | CAMKK2 | na | 20004 | -0.028 | -0.3112 | No |
| 30 | MAPK8 | na | 20414 | -0.033 | -0.3199 | No |
| 31 | IKBKG | na | 20820 | -0.038 | -0.3280 | No |
| 32 | NFKBIB | na | 22042 | -0.052 | -0.3583 | No |
| 33 | CPT1C | na | 22126 | -0.053 | -0.3557 | No |
| 34 | TNFRSF1A | na | 22241 | -0.055 | -0.3539 | No |
| 35 | ADIPOR2 | na | 22513 | -0.058 | -0.3563 | No |
| 36 | PRKAB2 | na | 23037 | -0.066 | -0.3652 | No |
| 37 | G6PC2 | na | 23090 | -0.067 | -0.3604 | No |
| 38 | ACSL4 | na | 23152 | -0.068 | -0.3558 | No |
| 39 | CD36 | na | 24229 | -0.084 | -0.3790 | Yes |
| 40 | ACSL6 | na | 24238 | -0.084 | -0.3713 | Yes |
| 41 | JAK2 | na | 24375 | -0.087 | -0.3671 | Yes |
| 42 | RXRB | na | 24595 | -0.091 | -0.3648 | Yes |
| 43 | PRKAB1 | na | 25012 | -0.099 | -0.3676 | Yes |
| 44 | PCK1 | na | 25015 | -0.099 | -0.3584 | Yes |
| 45 | TNFRSF1B | na | 25465 | -0.107 | -0.3614 | Yes |
| 46 | SLC2A4 | na | 25955 | -0.116 | -0.3646 | Yes |
| 47 | ACSL3 | na | 26206 | -0.121 | -0.3605 | Yes |
| 48 | SLC2A1 | na | 26243 | -0.122 | -0.3501 | Yes |
| 49 | STAT3 | na | 26362 | -0.124 | -0.3419 | Yes |
| 50 | NFKB1 | na | 26534 | -0.128 | -0.3348 | Yes |
| 51 | PPARGC1A | na | 27305 | -0.147 | -0.3432 | Yes |
| 52 | ACSL5 | na | 28542 | -0.181 | -0.3619 | Yes |
| 53 | NFKBIA | na | 28570 | -0.182 | -0.3457 | Yes |
| 54 | LEPR | na | 28912 | -0.192 | -0.3375 | Yes |
| 55 | RELA | na | 29651 | -0.214 | -0.3388 | Yes |
| 56 | NFKBIE | na | 30232 | -0.234 | -0.3335 | Yes |
| 57 | PRKAA2 | na | 30435 | -0.243 | -0.3166 | Yes |
| 58 | POMC | na | 31671 | -0.305 | -0.3237 | Yes |
| 59 | TNF | na | 31738 | -0.308 | -0.2967 | Yes |
| 60 | IRS2 | na | 31995 | -0.322 | -0.2739 | Yes |
| 61 | SOCS3 | na | 32238 | -0.337 | -0.2493 | Yes |
| 62 | PCK2 | na | 32780 | -0.374 | -0.2299 | Yes |
| 63 | RXRG | na | 33623 | -0.467 | -0.2104 | Yes |
| 64 | NPY | na | 34313 | -0.618 | -0.1723 | Yes |
| 65 | AKT3 | na | 34657 | -0.880 | -0.0998 | Yes |
| 66 | IRS1 | na | 34713 | -1.087 | 0.0004 | Yes |
Table: GSEA details [plain text format]

  

Fig 2: KEGG\_ADIPOCYTOKINE\_SIGNALING\_PATHWAY      
 Blue-Pink O' Gram in the Space of the Analyzed GeneSet

  

Fig 3: KEGG\_ADIPOCYTOKINE\_SIGNALING\_PATHWAY: Random ES distribution      
 Gene set null distribution of ES for **KEGG\_ADIPOCYTOKINE\_SIGNALING\_PATHWAY**
